# Supplementary material for: Ge Epitaxy at Ultralow Growth Temperatures Enabled by a Pristine Growth Environment
Source: ACS Appl Electron Mater. 2024 Dec 11;6(12):9029–39. doi: 10.1021/acsaelm.4c01678 (PMC11673087; doi:10.1021/acsaelm.4c01678)
Supplement: Supplementary file 1 — el4c01678_si_001.pdf [file el4c01678_si_001.pdf]

Supporting Information

Ge epitaxy at ultra-low growth temperatures enabled by a pristine growth environment

Christoph Wilflingseder[a], Johannes Aberl[a], Enrique Prado Navarrete[a], Günter Hesser[b], Heiko Groiss[b], Maciej O. Liedke[c], Maik Butterling[c], Andreas Wagner[c], Eric Hirschmann[c], Cedric Corley-Wiciak[d], Marvin H. Zoellner[e], Giovanni Capellini[e, f], Thomas Fromherz[a], Moritz Brehm[a]*

[a]Institute of Semiconductor and Solid State Physics, Johannes Kepler University Linz, Altenberger Straße 69, 4040, Linz, Austria

[b]Christian Doppler Laboratory for Nanoscale Phase Transformations, Center for Surface And Nanoanalytics (ZONA), Johannes Kepler University Linz, Altenberger Straße 69, 4040, Linz, Austria

[c]Helmholtz-Zentrum Dresden-Rossendorf e.V., Institute of Radiation Physics, Dresden, 01328, Germany

[d]ESRF – European Synchrotron Radiation Facility, 71, Avenue des Martyrs, CS 40220, 38043 Grenoble Cedex 9, France

[e]IHP – Leibniz-Institut für innovative Mikroelektronik, Im Technologiepark 25, D-15236, Frankfurt(Oder), Germany

[f] Dipartimento di Scienze, Università Roma Tre, V.le G. Marconi 446, 00146 Roma, Italy

[*]Email: christoph.wilflingseder@jku.at

As the growth pressure p_{Ge} is a critical parameter for ULT deposition, the growth chamber underwent a series of extensive preparations, including chamber conditioning, degassing, pumping, and the implementation of several gettering strategies. As a consequence, UHV pressure was achieved and the log files depicted in Figure S. 1 (a) and (b) present the lowest and the highest p_{Ge} observed during Ge deposition. Additionally, the shutter status, T_Gs, and growth rates are also included. The reference values for p_{Ge} are taken when the deposition of half the total Ge layer has been completed and are approximately ~$9\cdot10^{-11}$ mbar and ~$2\cdot10^{-10}$ mbar.

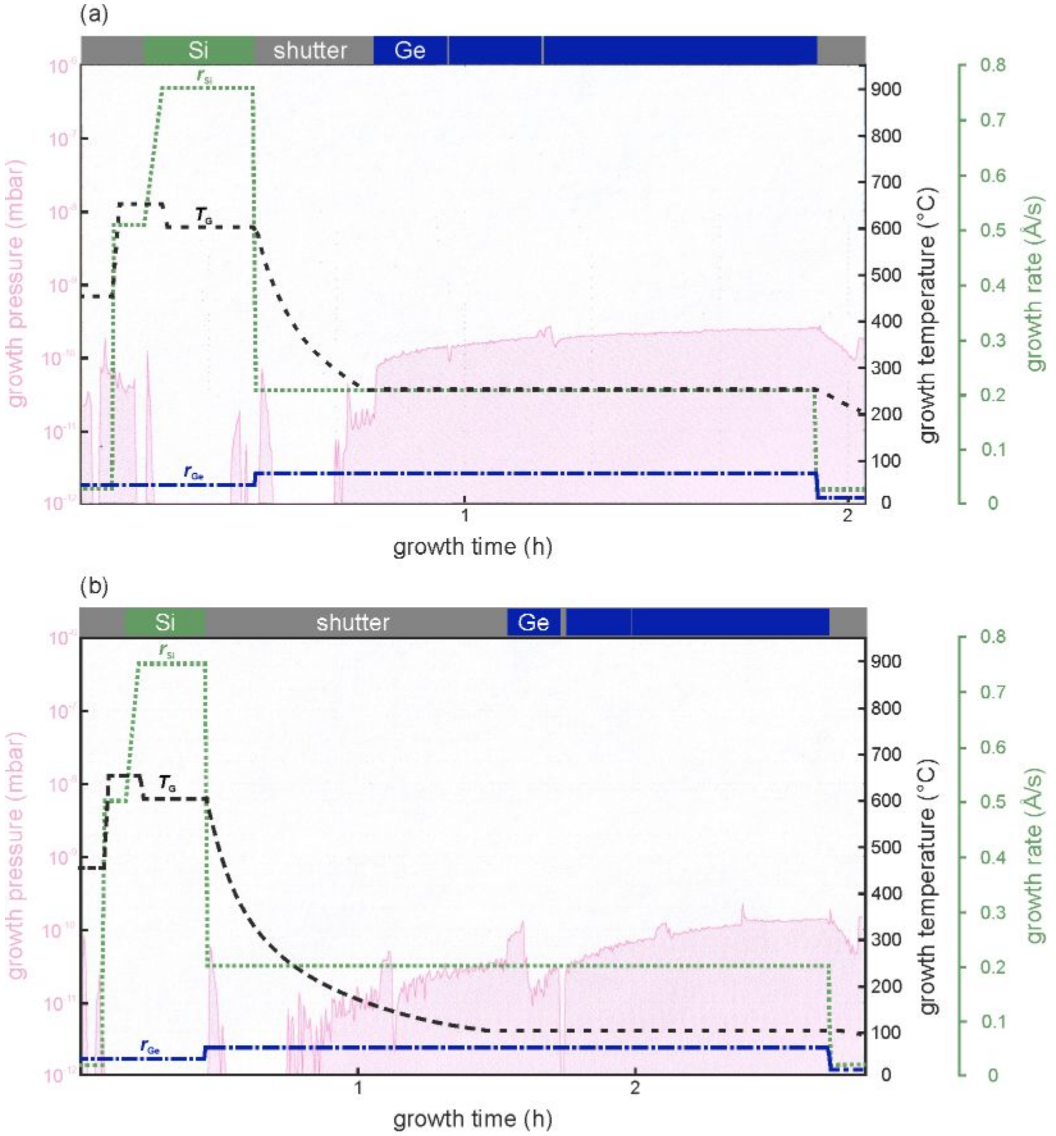

Figure S. 1. Growth log files. (a) 8 nm, 12 nm, and 16 nm at a growth temperature T_{Ge}=250˚C. (b) 8 nm, 12 nm und 16 nm Ge at T_{Ge}=100˚C.

In addition to the Ge/Ge(001) and Ge/Si(001) series, two complementary samples were grown. One sample was prepared by regrowing only the high-T, 75.5 nm thick Si buffer layer used in the heteroepitaxy study, and the resulting surface topography is shown in Figure S. 2(a). The vicinal surface of the Si(001) substrate was replicated. In the second sample, another Si homoepitaxy was conducted, with 50 nm of Si deposited at 200˚C on a 50 nm thick Si buffer with a growth temperature of 650˚C. As observed previously, ripples appeared in a regular pattern along the <100> direction, exhibiting a periodicity of approximately 2.4 μm^{-1} (see Figure S. 2(b)).

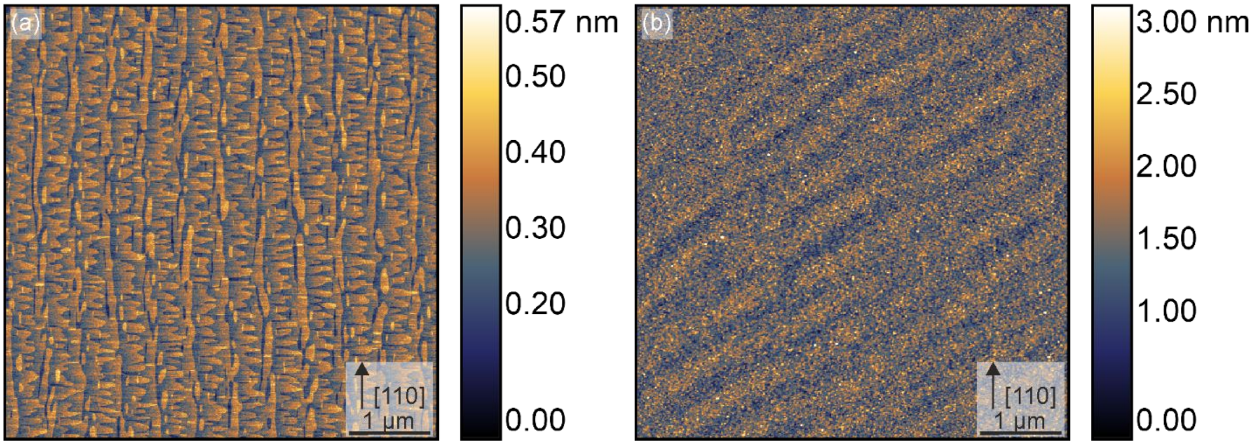

Figure S. 2. Complementary AFM images. (a) 5×5 µm^2 micrograph of the high temperature Si buffer. (b) 5×5 µm^2 micrograph of 50 nm Si homoepitaxy grown at 200˚C.

Figure S. 3 provides AFM micrographs with dimensions of 5×2.5 µm^2, which were recorded to investigate larger features. The ripples occur very regularly and exhibit a preferential orientation, occurring either along the [100] or [010] crystal direction. This finding aligns with previous research, which also demonstrated that the ripples are oriented along these directions[1,2]. Qualitatively, the ripples diminish for a growth temperature T_{Ge} = 300˚C and Ge coverages $\Theta_{Ge} \geq$ 8 nm. Given the numerous ripples present in these micrographs, they were used for the analysis of the periodicity.

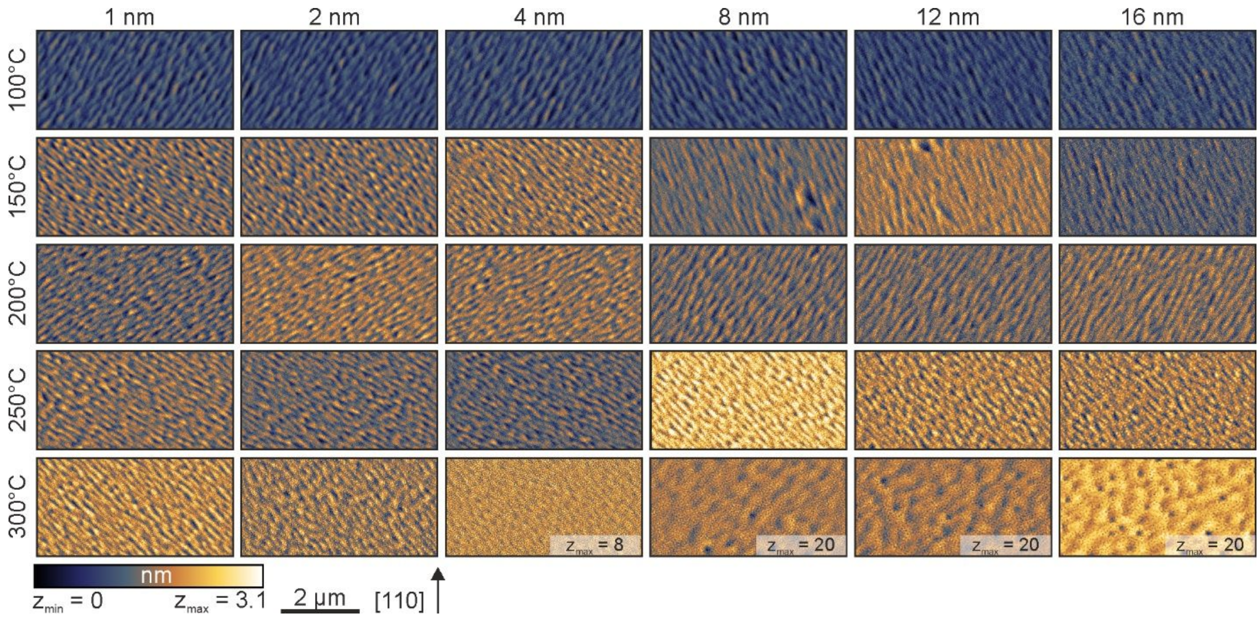

Figure S. 3. 5×2.5 µm² AFM images of the grown matrix.

Typically, high T_{Ge} > 400˚C is employed to ensure the growth of Ge on Si with good crystallinity. For ultra-low T_{Ge} (ULT ≡ <300˚C for Ge on Si), the ad-atom surface diffusion on Si and the Ge WL at low T_{Ge} is reduced, and impurities originating from the chamber background and the sources can be incorporated during growth[3]. Therefore, Figure S. 4 emphasizes the importance of stringent vacuum conditions. In deep UHV, the T_{Ge} exhibits minimal influence on the crystallinity (see Figure S. 4(a))). In contrast, higher growth pressures lead to an enhanced impingement rate of residual gases[4] (see Figure S. 4(c)). These impurities cannot be desorbed[3] and the results are amorphization or defective growth, which is depicted in Figure S. 4(b).

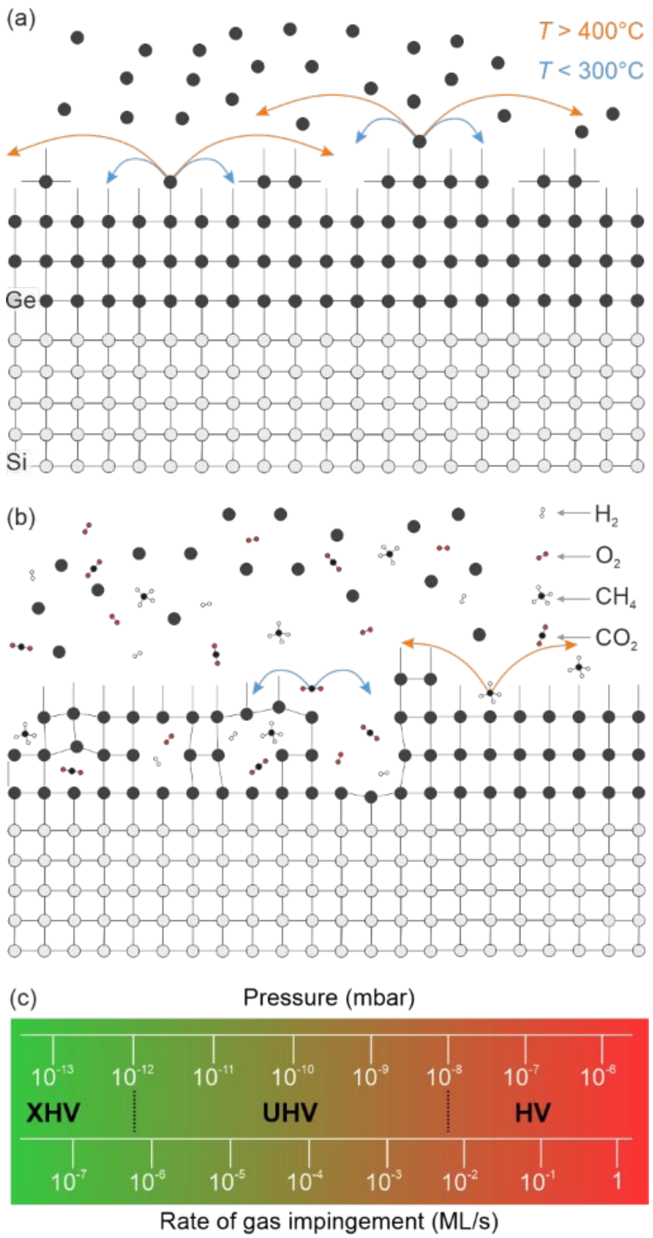

Figure S. 4 Scheme of the growth of Ge on Si at low T_{Ge}. The arrows indicate the surface diffusion at higher T_{Ge} (orange) and lower T_{Ge} (blue). (a) High epitaxial quality growth due to deep UHV growth conditions, i.e., mobility-limited growth. (b) Epitaxial growth at higher pressures, i.e., impurity-limited growth. Depending on the T_{Ge} and the possibility of impurity desorption or the lack thereof, crystalline growth or amorphization occurs. (c) Relationship between growth pressure and rate of gas impingement (ML/s), see Ref. [4].

REFERENCES

(1) Cullis, A. G.; Robbins, D. J.; Barnett, S. J.; Pidduck, A. J. Growth ripples upon strained SiGe epitaxial layers on Si and misfit dislocation interactions. *Journal of Vacuum Science & Technology A: Vacuum, Surfaces, and Films* **1994**, *12* (4), 1924–1931.

(2) Albrecht, M.; Christiansen, S.; Michler, J.; Dorsch, W.; Strunk, H. P.; Hansson, P. O.; Bauser, E. Surface ripples, crosshatch pattern, and dislocation formation: Cooperating mechanisms in lattice mismatch relaxation. *Applied Physics Letters* **1995**, *67* (9), 1232–1234. DOI: 10.1063/1.115017.

(3) Yabumoto, N. Analysis of molecular adsorbates on Si surfaces with thermal desorption spectroscopy. *AIP Conference Proceedings* **2009**, *449* (1), 696–701. DOI: 10.1063/1.56895.

(4) Farrow, R. F. C. *Molecular beam epitaxy: Applications to Key Materials*; Noyes Publications, 1995.
